# Time-to-antibiotics and clinical outcomes in patients with sepsis and septic shock: a prospective nationwide multicenter cohort study

**DOI:** 10.1186/s13054-021-03883-0

**Published:** 2022-01-13

**Authors:** Yunjoo Im, Danbee Kang, Ryoung-Eun Ko, Yeon Joo Lee, Sung Yoon Lim, Sunghoon Park, Soo Jin Na, Chi Ryang Chung, Mi Hyeon Park, Dong Kyu Oh, Chae-Man Lim, Gee Young Suh, the Korean Sepsis Alliance (KSA) investigators, Chae-Man Lim, Sang-Bum Hong, Dong Kyu Oh, Gee Young Suh, Kyeongman Jeon, Ryoung-Eun Ko, Young-Jae Cho, Yeon Joo Lee, Sung Yoon Lim, Sunghoon Park, Jeongwon Heo, Jae-myeong Lee, Kyung Chan Kim, Yeon Joo Lee, Youjin Chang, Kyeongman Jeon, Sang-Min Lee, Chae-Man Lim, Suk-Kyung Hong, Woo Hyun Cho, Sang Hyun Kwak, Heung Bum Lee, Jong-Joon Ahn, Gil Myeong Seong, Song-I. Lee, Sunghoon Park, Tai Sun Park, Su Hwan Lee, Eun Young Choi, Jae Young Moon

**Affiliations:** 1 Division of Pulmonary and Critical Care Medicine, Department of Medicine, Samsung Medical Center, Sungkyunkwan University School of Medicine, Seoul, Republic of Korea; 2 Department of Clinical Research Design and Evaluation, Samsung Advanced Institute for Health Sciences & Technology (SAIHST), Sungkyunkwan University, Seoul, Republic of Korea; 3 Department of Critical Care Medicine, Samsung Medical Center, Sungkyunkwan University School of Medicine, Seoul, Republic of Korea; 4 Department of Pulmonary and Critical Care Medicine, Seoul National University Bundang Hospital, Seongnam, Republic of Korea; 5 Department of Pulmonary, Allergy and Critical Care Medicine, Hallym University Sacred Heart Hospital, Anyang, Republic of Korea; 6 Department of Pulmonary and Critical Care Medicine, Asan Medical Center, University of Ulsan College of Medicine, Seoul, Republic of Korea; 7 Department of Critical Care Medicine, Division of Pulmonary and Critical Care Medicine, Department of Medicine, Samsung Medical Center, Sungkyunkwan University School of Medicine, 81 Irwon-ro, Gangnam-gu, Seoul, 06351 Republic of Korea

**Keywords:** Sepsis, Septic shock, Time-to-antibiotics, Hour-1 bundle, Mortality

## Abstract

**Background:**

Timely administration of antibiotics is one of the most important interventions in reducing mortality in sepsis. However, administering antibiotics within a strict time threshold in all patients suspected with sepsis will require huge amount of effort and resources and may increase the risk of unintentional exposure to broad-spectrum antibiotics in patients without infection with its consequences. Thus, controversy still exists on whether clinicians should target different time-to-antibiotics thresholds for patients with sepsis versus septic shock.

**Methods:**

This study analyzed prospectively collected data from an ongoing multicenter cohort of patients with sepsis identified in the emergency department. Adjusted odds ratios (ORs) were compared for in-hospital mortality of patients who had received antibiotics within 1 h to that of those who did not. Spline regression models were used to assess the association of time-to-antibiotics as continuous variables and increasing risk of in-hospital mortality. The differences in the association between time-to-antibiotics and in-hospital mortality were assessed according to the presence of septic shock.

**Results:**

Overall, 3035 patients were included in the analysis. Among them, 601 (19.8%) presented with septic shock, and 774 (25.5%) died. The adjusted OR for in-hospital mortality of patients whose time-to-antibiotics was within 1 h was 0.78 (95% confidence interval [CI] 0.61–0.99; *p* = 0.046). The adjusted OR for in-hospital mortality was 0.66 (95% CI 0.44–0.99; *p* = 0.049) and statistically significant in patients with septic shock, whereas it was 0.85 (95% CI 0.64–1.15; *p* = 0.300) in patients with sepsis but without shock. Among patients who received antibiotics within 3 h, those with septic shock showed 35% (*p* = 0.042) increased risk of mortality for every 1-h delay in antibiotics, but no such trend was observed in patients without shock.

**Conclusion:**

Timely administration of antibiotics improved outcomes in patients with septic shock; however, the association between early antibiotic administration and outcome was not as clear in patients with sepsis without shock.

## Take-home message

Timely administration of antibiotics improved outcomes in patients with septic shock. However, the association between early antibiotic administration and outcome was not as clear in patients with sepsis without shock.

## Introduction

Sepsis is a life-threatening syndrome characterized by physiological, pathological, and biochemical abnormalities that are induced by infection and associated with multiorgan failure and high mortality [1]. Compelling evidence has shown that delay in the initiation of appropriate antibiotic therapy is a risk factor for mortality; therefore, administration of antibiotics is recognized as a key component in the early treatment of sepsis [2–7]. In this regard, antibiotic administration has been included in the hour-1 bundle of the previous Surviving Sepsis Campaign guidelines, and the implementation of the hour-1 bundle was highly recommended to reduce mortality and morbidity [8, 9]. Indeed, several multinational studies reported that compliance to the Surviving Sepsis Campaign bundle was associated with mortality in patients with sepsis [10, 11].

Nevertheless, considerable controversy still exists regarding the association between the time of antibiotic administration and clinical outcomes in patients with sepsis/septic shock, and whether the administration of antibiotics within 1 h could improve outcomes in patients with sepsis [12, 13]. The Infectious Diseases Society of America recommends that the aggressive administration of antibiotics within 1 h might not be beneficial in sepsis [14] and may result in unintentional exposure to broad-spectrum antibiotics [15]. The American College of Emergency Physicians also noted a lack of evidence to recommend a strict time threshold for antibiotic administration in cases with sepsis [16]. In addition, organizing timely administration of antibiotics requires considerable effort and resources, and it may not be feasible to administer antibiotics within 1 h of presentation in all patients with sepsis [17].

Thus, this study aimed to evaluate the impact of time-to-antibiotics on in-hospital mortality in patients with sepsis. Furthermore, subgroup analysis was performed to assess whether the effect of time-to-antibiotics was significantly different between the sepsis and septic shock groups.

## Methods

### Study design and population

This prospective cohort study used data from an ongoing nationwide cohort of the Korean Sepsis Alliance. Patients were enrolled from 19 participating hospitals between September 2019 and December 2020. The protocols for patient enrollment and data collection have been described previously [18]. Patients were included if they were  19 years old and diagnosed with sepsis or septic shock in the emergency department. The diagnoses of sepsis and septic shock were based on the third International Consensus Definitions for Sepsis and Septic Shock (Sepsis-3) [1]. Patients were excluded if they were not admitted to hospital wards or the intensive care unit (ICU), not prescribed antibiotics, prescribed antibiotics that was not in accordance with guidelines, prescribed antibiotics for which the cultured organism proved to be resistant [8], or if their antibiotics were prescribed more than 12 h after time zero.

The study was approved by the institutional review boards of each participating hospital, and the requirement for informed consent was waived because of the noninterventional observational nature of the study.

### Clinical data collection

Data on demographic characteristics, coexisting conditions, severity of illness, treatment, and clinical outcomes were collected. These variables included demographic factors, such as age, sex, body mass index, comorbidities, Charlson comorbidity index score, history of antibiotic administration or hospitalization for two or more days within the past 90 days before presenting to the emergency department, Clinical Frailty Scale, admission source (e.g., other hospitals, skilled nursing facility, or home), measures of illness severity using the Sequential Organ Failure Assessment (SOFA) score [19], recognition of sepsis by physicians in the emergency department, infection data including site of infection (e.g., respiratory, abdominal, urinary, or skin/soft tissue), identification of pathogen, treatment data including sepsis bundle compliance, appropriateness of antibiotics, and time to administration of antibiotics, and clinical outcomes, including length of in-hospital stay, in-hospital mortality, and admission/transfer to the ICU.

Time-to-antibiotics was calculated as the time interval from time zero, defined as the time of triage in the emergency department to the time of antibiotic administration. Physicians were considered to have recognized sepsis if the diagnosis of sepsis was included in the differential diagnosis list in the medical records.

### Statistical analysis

Participants’ baseline characteristics were summarized as numbers and proportions for categorical variables and mean with standard deviation or median with interquartile range (IQR, 25th–75th percentiles) for continuous variables. Preliminary analysis was performed to compare the baseline characteristics and outcomes between patients who received antibiotics within 1 h and those after 1 h, using the Chi-square or Fisher’s exact tests for categorical variables and the Mann–Whitney U test for continuous variables.

Odds ratios (ORs) with 95% confidence intervals (CIs) for in-hospital mortality were calculated using a conditional logistic regression model, considering between-center differences as a stratification factor. To control for other potential confounding factors, age, sex, Charlson comorbidity index score, history of antibiotic prescription or hospitalization for two or more days within the past 90 days before presenting to the emergency department, Clinical Frailty Scale, recognition of sepsis by physicians in the emergency department, initial SOFA score, diagnosis of sepsis or septic shock, site of infection, identification of pathogen, and admission/transfer to the ICU were adjusted.

Landmark analyses among patients who were alive for more than 3 h after the diagnosis of sepsis or septic shock were performed to avoid survivor treatment selection bias. In addition, time-to-antibiotics was modeled as a continuous variable using restricted cubic splines with knots at the 5th, 35th, 65th, and 95th percentiles of the sample distribution to provide a flexible estimate of the dose–response relationship between time-to-antibiotics and in-hospital mortality. In particular, the association between in-hospital mortality and time as a continuous variable was evaluated independently in patients receiving antibiotics within 3 h, 3 h to 6 h, and after 6 h in patients with and without septic shock.

To assess the heterogeneity of associations between administration of broad-spectrum antibiotics within an hour and in-hospital mortality, additional analyses were performed by prespecified clinically relevant subgroups defined by median age (< 75 vs. ≥ 75 years), sex, body mass index (< 25 vs. ≥ 25 kg/m^2^), Charlson comorbidity index score (< 9 vs. ≥ 9) [20], history of antibiotic prescription or hospitalization for two or more days within the past 90 days before presentation to the emergency department, median initial SOFA score (< 6 vs. ≥ 6), recognition of sepsis by physicians in the emergency department, site of infection (respiratory or abdominal), identification of pathogen, and admission/transfer to the ICU. The interaction of time-to-antibiotics within 1 h with clinical characteristics was evaluated using Wald tests for cross-product terms in regression models.

All tests were two-sided, and a *p*-value < 0.05 was considered statistically significant. All analyses were performed using SAS® Visual Analytics (SAS Institute Inc., USA) and STATA (version 14; StataCorp LP, College Station, TX, USA).

## Results

### Study population

**Fig. 1:**
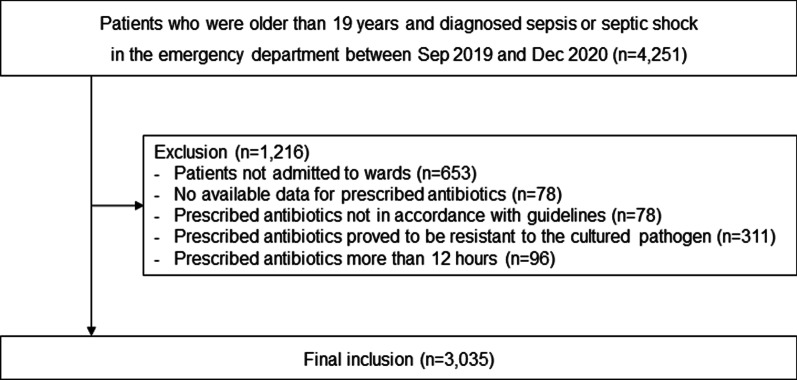
Flowchart of study participants

A total of 4251 patients were diagnosed with sepsis or septic shock in the emergency department between September 2019 and December 2020. Patients who were not admitted to wards from the emergency room (*n* = 653), had no available data for prescribed antibiotics (*n* = 78), were prescribed antibiotics that was not in accordance with guidelines (*n* = 78), were prescribed antibiotics proved to be resistant to the cultured pathogen (*n* = 311), or had received antibiotics more than 12 h after time zero (*n* = 96) were excluded. Consequently, 3035 patients were included in the analyses (Fig. 1).

### Baseline characteristics of patients

The mean (standard deviation) age of study patients was 71.3 (13.5) years, 57.5% were men, and 601 (19.8%) were diagnosed with septic shock. The median time-to-antibiotics was 141 min (IQR, 80–230 min), and 512 (16.9%) patients received antibiotics within 1 h.

Compared to patients who did not receive antibiotics within 1 h, those who received antibiotics within 1 h were more likely to have a history of taking antibiotics within the past 90 days (14.4% vs. 20.1%, *p* < 0.01), had a higher initial SOFA score (5 vs. 6, *p* < 0.01), and were diagnosed with septic shock (17.3% vs. 32.0%, *p* < 0.01) (Table 1).

**Table 1:** Baseline characteristics of study participants according to time-to-antibiotics < 1 h or > 1 h (*n* = 3035)

Variables	Administration of broad-spectrum antibiotics in 1 h		*p*-value
	No (*n* = 2523)	Yes (*n* = 512)	
*Age, years*	74 (63–81)	75 (65–81)	0.12
*Sex, male*	1448 (57.4)	298 (58.2)	0.74
*Body mass index, kg/m*^*2*^	21.8 (4.2)	22.0 (4.1)	0.41
*Comorbidity*			
Diabetes mellitus	966 (38.2)	200 (39.0)	0.75
History of myocardial infarction	259 (10.3)	51 (9.9)	0.83
Congestive heart failure	190 (7.5)	30 (5.9)	0.18
Chronic neurological disease	495 (19.6)	88 (17.2)	0.20
Chronic liver disease	280 (11.1)	51 (9.9)	0.68
Chronic kidney disease	419 (16.6)	73 (14.2)	0.19
Connective tissue disease	63 (2.5)	13 (2.5)	0.96
Solid malignant tumors	474 (18.8)	99 (19.3)	0.78
*Charlson comorbidity index score*	5 (4–7)	5 (4–7)	0.14
≥ 9	336 (13.2)	70 (13.7)	0.83
*History of antibiotic prescription within the past 90 days*			** < 0.01**
No	2070 (82.1)	390 (76.2)	
Yes	364 (14.4)	103 (20.1)	
Unknown	89 (3.5)	19 (3.7)	
*Hospitalization for two or more days within the past 90 days*	1043 (41.3)	208 (40.6)	0.77
*Clinical Frailty Scale*			0.11
Very fit	99 (3.9)	27 (5.3)	
Well	186 (7.4)	36 (7)	
Managing well	379 (15)	54 (10.5)	
Vulnerable	393 (15.6)	79 (15.4)	
Mildly frail	264 (10.5)	50 (9.8)	
Moderately frail	328 (13)	60 (11.7)	
Severely frail	479 (19)	115 (22.5)	
Very severely frail	380 (15.1)	86 (16.8)	
Terminally ill	15 (0.6)	5 (1)	
*Initial SOFA score*	5 (4–8)	6 (4–9)	** < 0.01**
*Septic shock*	437 (17.3)	164 (32.0)	** < 0.01**
*Recognition of sepsis by physicians in the emergency department*	943 (37.4)	234 (45.7)	** < 0.01**
Site of infection*			
Respiratory	1199 (47.5)	259 (50.6)	0.21
Abdominal	660 (26.2)	143 (27.9)	0.41
Urinary	131 (5.2)	31 (6.1)	0.43
Skin/soft tissue	90 (3.6)	13 (2.5)	0.24
*Type of infection*			0.16
Community	1673 (66.3)	324 (63.3)	
Nursing home-acquired	179 (7.1)	29 (5.7)	
Nursing hospital-acquired	331 (13.1)	75 (14.6)	
Hospital-acquired	340 (13.5)	84 (16.4)	
*Identification of pathogen*	1380 (54.7)	308 (60.2)	**0.02**
*ICU admission/transfer*	1205 (47.8)	288 (56.3)	** < 0.01**
*Length of hospital stay, days*	12 (6–20)	11 (6–19.5)	0.35

Bold values indicate parameters that are statistically significant

Data are presented as mean (SD), median (interquartile range), or number (%)

ER, emergency room; ICU, intensive care unit; SOFA, Sequential Organ Failure Assessment

^*^Mutually nonexclusive

### In-hospital mortality

A total of 774 patients (25.5%) died during the study period. No significant differences were found in unadjusted in-hospital mortality between patients who received antibiotics within 1 h and those who did not receive antibiotics within 1 h (25.7% vs. 24.6%, *p* = 0.61).

However, in the adjusted analysis, the OR for in-hospital mortality of patients with time-to-antibiotics within 1 h was 0.78 (95% CI 0.61–0.99; *p* = 0.046) compared to those with time-to-antibiotics not within 1 h. In subgroup analyses, the adjusted OR for in-hospital mortality in patients with sepsis without shock with time-to-antibiotics within 1 h was 0.85 (95% CI 0.64–1.15; *p* = 0.300). In patients with septic shock, the adjusted OR for in-hospital mortality of patients with time-to-antibiotics within 1 h was 0.66 (95% CI 0.44–0.99; *p* = 0.049). The association between time-to-antibiotics within 1 h and in-hospital mortality in the landmark analysis, confined to patients who survived more than 3 h, showed similar results to the primary analysis (Table 2).

**Table 2:** Risk-adjusted odds ratios (95% confidence interval) for in-hospital mortality associated with administration of broad-spectrum antibiotics in 1 h

In-hospital mortality	Administration of broad-spectrum antibiotics in 1 h		*p*-value
	No	Yes OR (95% CI)*	
*All participants (n* = *3035)*			
Overall	*Reference*	**0.78 (0.61–0.99)**	**0.046**
Without septic shock	*Reference*	0.85 (0.64**–**1.15)	0.300
With septic shock	*Reference*	**0.66 (0.44–0.99)**	**0.049**
*Landmark analysis (N* = *3018)*			
Overall	*Reference*	**0.78 (0.61–0.99)**	**0.046**
Without septic shock	*Reference*	0.86 (0.64**–**1.15)	0.310
With septic shock	*Reference*	**0.65 (0.43–0.98)**	**0.042**

Bold values indicate parameters that are statistically significant

^*^ To control for other potential confounding factors, age, sex, Charlson comorbidity index score (< 9 vs. ≥ 9), history of antibiotic prescription or hospitalization for two or more days within the past 90 days before presenting to the emergency department, recognition of sepsis by physicians in the emergency department, Clinical Frailty Scale score, initial SOFA score, diagnosis (sepsis or septic shock), site of infection (pulmonary vs. abdominal), identification of pathogen, admission/transfer to ICU were adjusted

**Fig. 2:**
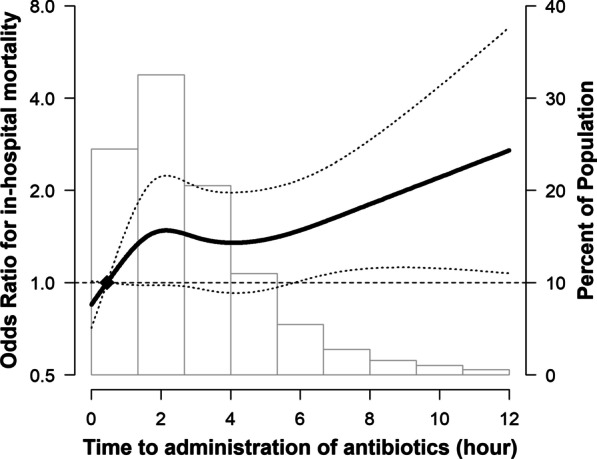
Estimated odds ratios (ORs) for in-hospital mortality by time-to-antibiotics with 95% confidence interval (CI)s. Solid line and long dashed lines represent OR and its 95% CIs

**Fig. 3:**
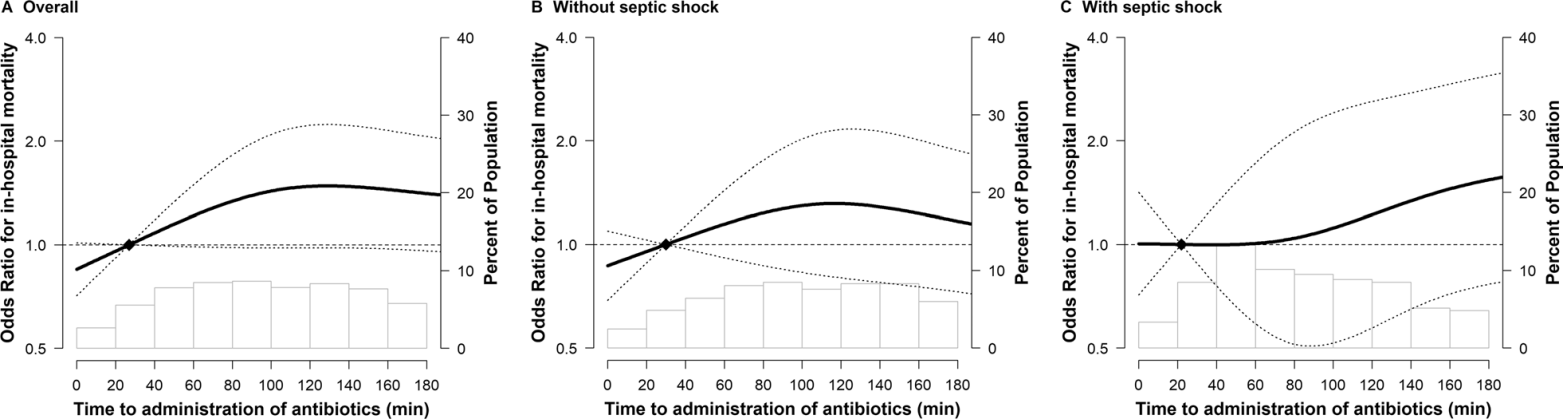
**a** Estimated odds ratios (ORs) for in-hospital mortality by time-to-antibiotics with 95% confidence intervals (CI), confined to patients with time-to-antibiotics within 3 h. **b** Estimated ORs for in-hospital mortality by time-to-antibiotics with 95% CIs, confined to patients without shock and time-to-antibiotics within 3 h. **c** Estimated ORs for in-hospital mortality by time-to-antibiotics with 95% CIs, confined to patients with shock and time-to-antibiotics within 3 h. Solid line and long dashed lines represent OR and its 95% CIs

In spline regression models, the association between time-to-antibiotics and in-hospital mortality was nonlinear (*p*-value for nonlinear spline terms = 0.02, Fig. 2), with a stronger association within 3 h of time-to-antibiotics than that after 3 h (Fig. 3a). Within 3 h, patients with septic shock showed 35% (OR 1.35; 95% CI 1.01–1.81; *p* = 0.042) increased risk of mortality by every 1-h delay in antibiotic administration (Fig. 3b), but this trend was not observed in patients without shock (OR 1.01; 95% CI 0.82–1.23; *p* = 0.94, Fig. 3c). Within the interval between 3 and 6 h and after 6 h, no statistically significant increasing trends were observed in the association between time delay in antibiotic administration as a continuous variable and mortality in both patients with and without septic shock.

### Subgroup analysis

**Fig. 4:**
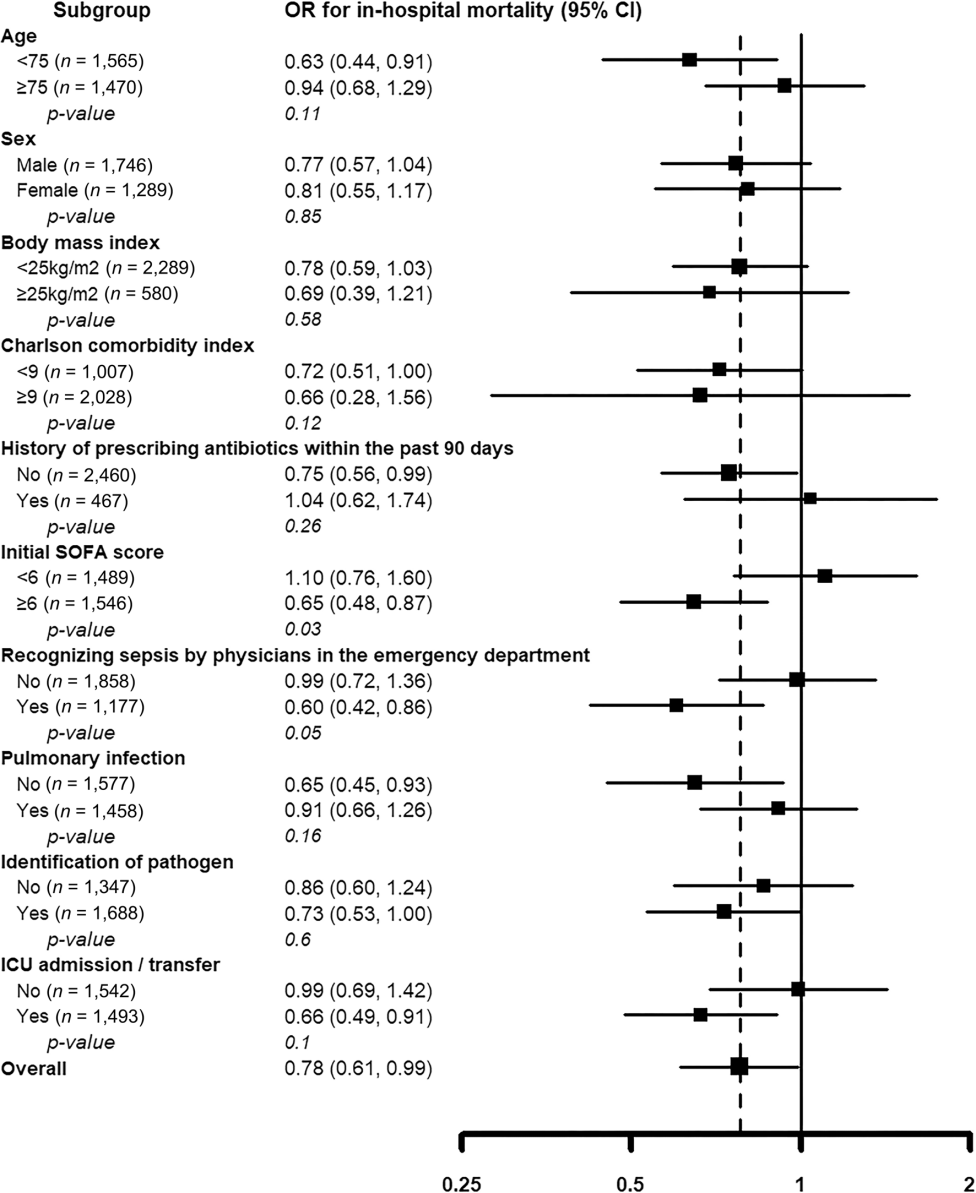
Risk-adjusted odds ratios (ORs) of in-hospital mortality by time-to-antibiotics in the prespecified subgroups for all study participants. Shown are ORs, with 95% confidence intervals, for in-hospital death for each hour of time-to-antibiotics

Subgroup analyses were performed to evaluate the influence of antibiotic administration within 1 h on in-hospital mortality among the various subgroups (Fig. 4). Administration of antibiotics within 1 h decreased in-hospital mortality in patients with an initial SOFA score > 6 (OR 0.65; 95% CI 0.48–0.87; *p* = 0.03) and those who were recognized as having sepsis by physicians in the emergency department (OR 0.60; 95% CI 0.42–0.86; *p* = 0.05). In addition, administration of antibiotics within 1 h reduced in-hospital mortality in patients who were younger than 75 years old (OR 0.63; 95% CI 0.44–0.91), did not have a history of antibiotic prescription within the past 90 days (OR 0.75; 95% CI 0.56–0.99), had non-pulmonary infection (OR 0.65; 95% CI 0.45–0.93), and admitted to the ICU (OR 0.64; 95% CI 0.47–0.89).

## Discussion

This multicenter prospective study assessed the impact of time to administration of antibiotics on mortality in patients with sepsis. For patients with septic shock, administration of broad-spectrum antibiotics within 1 h of sepsis recognition reduced in-hospital mortality. However, in patients with sepsis without shock, the association between the antibiotic administration within 1 h and in-hospital mortality was not statistically significant. In spline regression models, limited to patients who received antibiotics within 3 h, patients with septic shock showed an increased risk of mortality for every hour of delay in antibiotic administration, but no such trend was observed in those without shock.

One of our most notable findings was the different impact of time-to-antibiotics on mortality in patients with sepsis with and without shock. This finding supports the recent statements from the Infectious Diseases Society of America and American College of Emergency Physicians that emphasize the insufficient evidence of a strict time threshold in the administration of antibiotics in patients with sepsis and suggests that patients with septic shock might derive the greatest benefit from immediate antibiotic administration [15, 16]. In addition, these data are compatible with the most recent guidelines from the Surviving Sepsis Campaign, which recommends antibiotic administration within 1 h in patients with shock, but prioritizes rapid assessment of the likelihood of infection in patients with possible sepsis without shock [21].

The evidence supporting previous recommendations for administration of broad-spectrum antibiotics within 1 h in all patients with sepsis was mainly from studies on patients confined to septic shock or based on retrospective studies [2–4, 22, 23]. Two recent multicenter studies with a large sample sizes also support the findings of our study. In a study that investigated the effect of time to treatment on mortality of mandated emergency care for sepsis in 149 New York hospitals, the odds of in-hospital mortality were increased by 7% for every hour of delay in antibiotic administration in patients with septic shock, but not in those without shock [24]. In a retrospective analysis of 35,000 patients with sepsis admitted in the emergency department of 21 hospitals in Northern California, a delay in antibiotic administration was associated with increased odds of mortality, which was greatest in patients with septic shock [25]. However, in this study, an increased OR of mortality was observed in all sepsis severity strata. Notably, in this study, the definition of sepsis was based on administrative codes, with its inherent limitations. Another large multicenter study conducted in US showed that early administration of antibiotics was associated with reduced long-term mortality in sepsis patients identified using Sepsis-3 criteria [7]. But this study was designed retrospectively and mostly included less severe patients; only 7.3% of patients needed vasopressors within 24 h. In our study, sepsis was diagnosed using the Sepsis-3 criteria, and to the best of our knowledge, our study is the first to comprehensively evaluate the association between the time-to-antibiotic administration and mortality of patients with sepsis or septic shock classified according to the new diagnostic criteria in a large prospective multicenter cohort.

Aggressive treatment with rapid initiation of broad-spectrum antibiotics in all patients suspected of sepsis entails unnecessary exposure to antibiotics of a significant number of patients who do not need antibiotics together with the associated risk of adverse effects of antibiotics, increased level of antimicrobial resistance, increased economic burden, and adverse outcomes [14, 26, 27]. Moreover, most hospitals do not have the resources to administer antibiotics within 1 h to all patients with suspected sepsis. In fact, in a previous study that investigated the effect of time to treatment on mortality of mandated emergency care for sepsis in 149 New York hospitals, more than half of the patients with sepsis did not receive antibiotics within 3 h of sepsis onset despite the implementation of the severe sepsis and septic shock management bundle [22]. Selecting a subpopulation of patients who could benefit most from this intervention could help in prioritizing areas of improvement in the management of sepsis/septic shock.

In addition to the presence of shock, our study indicates that patients with several distinguishing characteristics might benefit from early antibiotic treatment. A significant reduction in in-hospital mortality was observed in patients who had higher SOFA scores or were admitted to the ICU due to early administration of antibiotics, suggesting that patients with clinically severe disease should receive antibiotics as soon as possible. Other factors associated with improved survival were younger age, non-pulmonary infection as the cause of sepsis, and no previous history of antibiotic treatment within 3 months. Further studies are needed to confirm if patients with these characteristics might benefit from the early administration of antibiotics. One interesting factor associated with improved outcome was the recognition of sepsis by the treating physician in the emergency room. This may also be a surrogate marker of patients’ disease severity because clinicians might be more inclined to give a diagnosis of sepsis to patients who are severely ill compared to just labeling them according to the site of infection. It would be interesting to identify if better education of emergency physicians on the recognition and treatment of sepsis might lead to better outcomes in patients with sepsis [28].

One of the strengths of our study is that biases associated with observational studies were reduced as much as possible. Patients who did not receive antibiotics or who did not receive appropriate antibiotics were excluded because the objective of the study was to examine the impact of early administration of appropriate antibiotic treatment on patient outcomes. In addition, the results were adjusted for all confounders thought to influence the outcome. Moreover, landmark analysis was performed as a sensitivity analysis to adjust for survivor treatment selection bias.

Potential limitations should be acknowledged to fully appreciate the results of our study. First, as this study was conducted only in patients from 19 centers in the Republic of Korea, the results might not be generalizable to different regions. All participating centers were university-affiliated with many tertiary referral centers. Second, although this study included more than 3000 patients, the generalizability of our findings was limited with a relatively small sample size. It might have underestimated the effect of intravenous antibiotic administration in 1 h to reduce mortality in patients without shock, and might not have the enough power to decipher small but important difference in specific subgroups. Third, this study included only patients who were diagnosed with sepsis at presentation to the emergency room. Thus, the results may not be generalizable to patients with sepsis in the hospital.

## Conclusion

Timely administration of antibiotics improved outcomes in patients with septic shock; however, the association between early antibiotic administration and outcome was not as clear in those with sepsis without shock. Further studies are warranted to investigate the relationship between time-to-antibiotics and adverse outcomes in patients with sepsis without shock.

## Data Availability

The datasets used and/or analyzed during the current study are available from the corresponding author upon reasonable request.

## Abbreviations

CI: Confidence intervals
ICU: Intensive care unit
IQR: Interquartile Range
OR: Odds ratio
OS: Overall survival
SOFA: Sequential Organ Failure Assessment

## References

[1] Singer M, Deutschman CS, Seymour CW, Shankar-Hari M, Annane D, Bauer M (2016). The third international consensus definitions for sepsis and septic shock (sepsis-3). JAMA.

[2] Ferrer R, Martin-Loeches I, Phillips G, Osborn TM, Townsend S, Dellinger RP (2014). Empiric antibiotic treatment reduces mortality in severe sepsis and septic shock from the first hour: results from a guideline-based performance improvement program. Crit Care Med.

[3] Kumar A, Roberts D, Wood KE, Light B, Parrillo JE, Sharma S (2006). Duration of hypotension before initiation of effective antimicrobial therapy is the critical determinant of survival in human septic shock. Crit Care Med.

[4] Gaieski DF, Mikkelsen ME, Band RA, Pines JM, Massone R, Furia FF (2010). Impact of time to antibiotics on survival in patients with severe sepsis or septic shock in whom early goal-directed therapy was initiated in the emergency department. Crit Care Med.

[5] Garnacho-Montero J, Aldabo-Pallas T, Garnacho-Montero C, Cayuela A, Jiménez R, Barroso S (2006). Timing of adequate antibiotic therapy is a greater determinant of outcome than are TNF and IL-10 polymorphisms in patients with sepsis. Crit Care.

[6] Liang SY, Kumar A (2015). Empiric Antimicrobial Therapy in Severe Sepsis and Septic Shock: Optimizing Pathogen Clearance. Current Infectious Disease Reports.

[7] Peltan ID, Brown SM, Bledsoe JR, Sorensen J, Samore MH, Allen TL (2019). ED door-to-antibiotic time and long-term mortality in sepsis. Chest.

[8] Rhodes A, Evans LE, Alhazzani W, Levy MM, Antonelli M, Ferrer R (2017). Surviving sepsis campaign: international guidelines for management of sepsis and septic shock: 2016. Crit Care Med.

[9] Levy MM, Evans LE, Rhodes A (2018). The surviving sepsis campaign bundle: 2018 update. Intensive Care Med.

[10] Rhodes A, Phillips G, Beale R, Cecconi M, Chiche JD, De Backer D (2015). The surviving sepsis campaign bundles and outcome: results from the International Multicentre Prevalence Study on Sepsis (the IMPreSS study). Intensive Care Med.

[11] Levy MM, Rhodes A, Phillips GS, Townsend SR, Schorr CA, Beale R (2015). Surviving Sepsis Campaign: association between performance metrics and outcomes in a 7.5-year study. Crit Care Med.

[12] Taylor SP, Anderson WE, Beam K, Taylor B, Ellerman J, Kowalkowski MA (2021). The association between antibiotic delay intervals and hospital mortality among patients treated in the emergency department for suspected sepsis. Crit Care Med.

[13] Puskarich MA, Trzeciak S, Shapiro NI, Arnold RC, Horton JM, Studnek JR (2011). Association between timing of antibiotic administration and mortality from septic shock in patients treated with a quantitative resuscitation protocol. Crit Care Med.

[14] Force IST (2018). Infectious Diseases Society of America (IDSA) position statement: why IDSA did not endorse the surviving sepsis campaign guidelines. Clin Infect Dis.

[15] Rhee C, Chiotos K, Cosgrove SE, Heil EL, Kadri SS, Kalil AC (2020). Infectious diseases society of america position paper: recommended revisions to the national severe sepsis and septic shock early management bundle (SEP-1) sepsis quality measure. Clin Infect Dis.

[16] Yealy DM, Mohr NM, Shapiro NI, Venkatesh A, Jones AE, Self WH (2021). Early care of adults with suspected sepsis in the emergency department and out-of-hospital environment: a consensus-based task force report. Ann Emerg Med.

[17] Barbash IJ, Davis BS, Yabes JG, Seymour CW, Angus DC, Kahn JM (2021). Treatment patterns and clinical outcomes after the introduction of the medicare sepsis performance measure (SEP-1). Ann Intern Med.

[18] Park S, Jeon K, Oh DK, Choi EY, Seong GM, Heo J (2020). Normothermia in patients with sepsis who present to emergency departments is associated with low compliance with sepsis bundles and increased in-hospital mortality rate. Crit Care Med.

[19] Vincent JL, Moreno R, Takala J, Willatts S, De Mendonça A, Bruining H (1996). The SOFA (Sepsis-related Organ Failure Assessment) score to describe organ dysfunction/failure. On behalf of the Working Group on Sepsis-Related Problems of the European Society of Intensive Care Medicine. Intensive Care Med.

[20] Zhao L, Leung LH, Wang J, Li H, Che J, Liu L (2017). Association between Charlson comorbidity index score and outcome in patients with stage IIIB-IV non-small cell lung cancer. BMC Pulm Med.

[21] Evans L, Rhodes A, Alhazzani W, Antonelli M, Coopersmith CM, French C (2021). Surviving sepsis campaign: international guidelines for management of sepsis and septic shock 2021. Intensive Care Med.

[22] Barie PS, Hydo LJ, Shou J, Larone DH, Eachempati SR (2005). Influence of antibiotic therapy on mortality of critical surgical illness caused or complicated by infection. Surg Infect (Larchmt).

[23] Ferrer R, Artigas A, Suarez D, Palencia E, Levy MM, Arenzana A (2009). Effectiveness of treatments for severe sepsis: a prospective, multicenter, observational study. Am J Respir Crit Care Med.

[24] Seymour CW, Gesten F, Prescott HC, Friedrich ME, Iwashyna TJ, Phillips GS (2017). Time to treatment and mortality during mandated emergency care for sepsis. N Engl J Med.

[25] Liu VX, Fielding-Singh V, Greene JD, Baker JM, Iwashyna TJ, Bhattacharya J (2017). The timing of early antibiotics and hospital mortality in sepsis. Am J Respir Crit Care Med.

[26] Patel JJ, Bergl PA (2019). COUNTERPOINT: should broad-spectrum antibiotics be routinely administered to all patients with sepsis as soon as possible?. No Chest.

[27] Nelson RE, Hatfield KM, Wolford H, Samore MH, Scott RD, Reddy SC (2021). National estimates of healthcare costs associated with multidrug-resistant bacterial infections among hospitalized patients in the United States. Clin Infect Dis.

[28] Kumar P, Jordan M, Caesar J, Miller S (2015). Improving the management of sepsis in a district general hospital by implementing the 'Sepsis Six' recommendations. BMJ Open Quality.

